# Vibrio fluvialis Bacteremia With Hemorrhagic Bullae: A Case Report

**DOI:** 10.7759/cureus.42612

**Published:** 2023-07-28

**Authors:** Thomas J Smith, Yama Osmanzai, Mohamed Faris

**Affiliations:** 1 Department of Internal Medicine, Grand Strand Medical Center, Myrtle Beach, USA; 2 Department of Infectious Disease, Coastal Carolina Infectious Disease, Myrtle Beach, USA; 3 Department of Internal Medicine, Grand Strand Regional Medical Center, Myrtle Beach, USA

**Keywords:** infectious diarrhea, foodborne illness, hemorrhagic bullae, vibrio fluvialis, gram-negative bacteremia

## Abstract

*V. fluvialis* is a pathogenic Gram-negative bacillus typically resulting in gastroenteritis in humans. It has recently been identified as a growing concern for public health. The case presented is an uncommon case of *V. fluvialis *causing bacteremia, as well as hemorrhagic skin lesions. Other reported cases have also highlighted unexpected manifestations, such as cerebritis, bacterial peritonitis, and otitis externa. These atypical presentations can happen in immunocompromised individuals. There are no established guidelines currently for the treatment of *V. fluvialis* bacteremia. This case presents *V.* *fluvialis* bacteremia that improved with doxycycline without the need for incision and drainage of the patient’s lower extremity lesions.

## Introduction

*Vibrio fluvialis* is a foodborne pathogenic bacteria that is implicated in gastroenteritis in humans after consumption of seafood. In cases of immunocompromised individuals, *V. fluvialis* can lead to bacteremia and wound infections, especially in situations of poor sanitation [1]. It is a motile halophilic Gram-negative bacillus and is considered an emerging pathogen in public health. The bacteria is found globally and inhabit brackish or seawater environments, although it can be isolated in animal feces, human feces, sewage, and seafood products [1]. The pathogen can often be difficult to identify due to its similarities with *Vibrio cholerae* and *Aeromonas* spp. [2]. The typical presentation of *V. fluvialis* infection includes symptoms such as watery bloody diarrhea, vomiting, abdominal cramping, and fever. In this particular case, the patient exhibited the aforementioned symptoms along with the presence of hemorrhagic bullae and bacteremia.

## Case presentation

A 64-year-old white male with a medical history of atrial fibrillation, congestive heart failure, hypertension, recurrent purulent cellulitis of his right lower extremity, and chronic anemia presented to the emergency department with a chief complaint of four days of watery diarrhea with the development of a subjective fever at home. He additionally had a right lower extremity wound, approximately 1 inch in diameter, with serosanguinous drainage. The patient was from a coastal state within the United States and was on vacation to another coastal state. His home medications included aspirin, carvedilol, hydroxyzine, furosemide, allopurinol, and potassium chloride. His surgical history included an atrioventricular node ablation for his atrial fibrillation. The patient denied any alcohol or illicit drug use and stated that he has never been a smoker.

**Figure 1:**
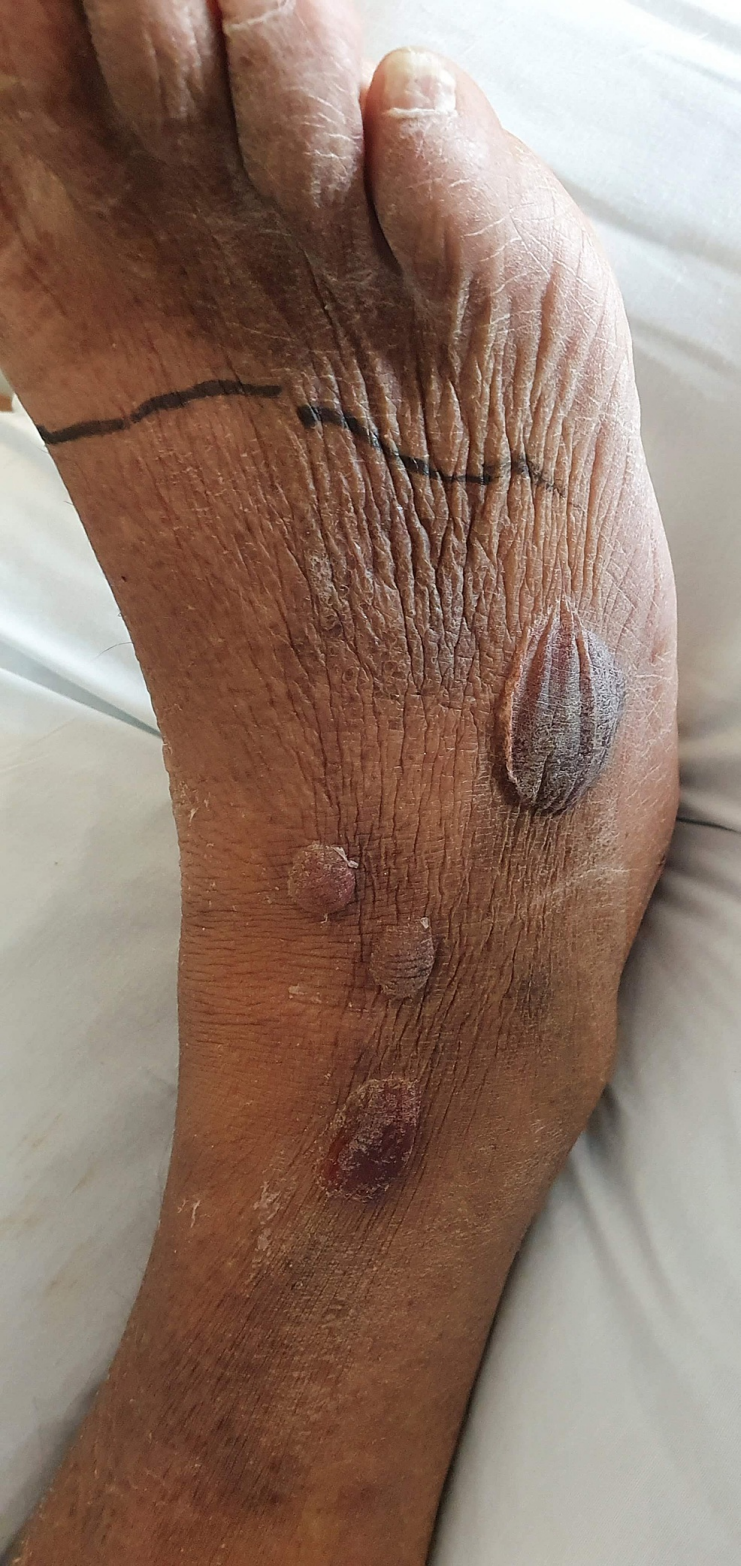
Flaccid hemorrhagic bullae on the right lower extremity before treatment.

In the emergency department, the patient was febrile at 104.1 °F, tachycardic with a heart rate of 111 beats per minute, and hypertensive. The initial lab work showed a non-anion gap metabolic acidosis. Lactic acid was normal (1.0 mmol/L), c-reactive protein was elevated (3.66 mg/dL), erythrocyte sedimentation rate was elevated (35 mm/hour), d-dimer was markedly elevated (>7,000 ng/mL), white blood cell count was normal (3.9 K/mm^3^), and hemoglobin was low (8.9 gm/dL) with microcytosis (77.4 fL). No prior laboratory studies were available for comparison. Blood and wound cultures were collected at this time. He was given one dose of cefazolin. On physical exam, hemorrhagic bullae of the lower extremity were noted without crepitus (Figure 1).

**Figure 2:**
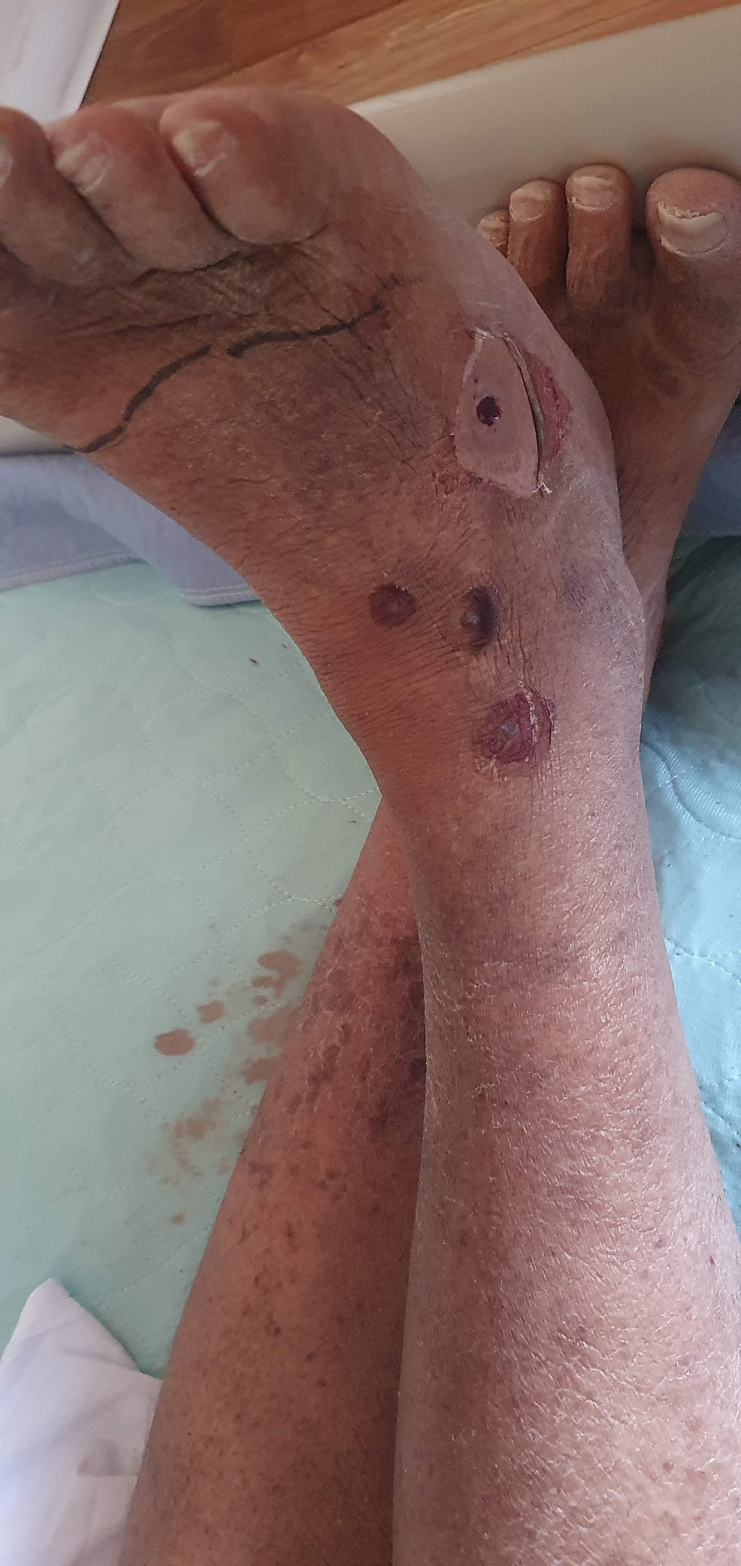
Resolution of hemorrhagic bullae within three days of treatment.

The patient was admitted for further management of sepsis suspected to be due to cellulitis versus infectious gastroenteritis/colitis. Stool polymerase chain reaction (PCR) testing was negative for Salmonella, Shigella, Campylobacter, and Enterotoxigenic *Escherichia coli*. He was initially started on empiric antibiotic therapy with vancomycin (renal dose-dependent) and piperacillin-tazobactam (3.375 gm three times daily intravenously) while blood and wound cultures were pending. COVID-19 PCR was negative. The differential diagnosis of the patient's hemorrhagic bullae included infectious causes such as herpes simplex or staphylococcal scalded skin syndrome, as well as autoimmune diseases such as pemphigus vulgaris. An ultrasound of his right lower extremity was obtained that showed a phlegmon or fluid collection measuring 1.9 cm × 1.3 cm × 0.6 cm that was 3 mm deep to the skin. General surgery was consulted for possible incision and drainage and evaluated the patient on hospital day two. A lower extremity computed tomography (CT) scan was obtained, which ruled out gas production. Due to his clinical improvement with antibiotics, surgical intervention and skin biopsy were deferred (Figure 2).

Blood cultures on the second day of admission grew anaerobic Gram-negative bacilli and wound cultures grew *E.* *coli *and *Staphylococcus aureus*. The infectious disease specialist was consulted, and the patient was continued on piperacillin-tazobactam, the vancomycin was discontinued, and daptomycin (950 mg/mL every 48 hours intravenously) was initiated. On day four of hospitalization, daptomycin was discontinued and doxycycline (100 mg intravenously twice daily) was started for concerns for Vibrio and/or Pasteurella. On the thirteenth day of admission, blood cultures speciated to *V. fluvialis*. The cultures were sent out to an outside lab and confirmed. After sensitivity results were reviewed (Table 1), the patient was started initially on ceftriaxone (2 g intravenously daily) and doxycycline (100 mg PO twice daily). The patient experienced oropharyngeal swelling, which led to a change in the medication from ceftriaxone to levofloxacin (750 mg intravenously daily). The patient's symptoms of soreness and neck swelling persisted, and as a result, the treatment was changed to doxycycline (100 mg intravenously twice daily). The patient had never reported any allergies to the aforementioned antibiotics, to the best of his knowledge.

**Table 1:** Sensitivity results.

Antibiotic	Susceptibility
Aztreonam	Sensitive
Cefepime	Sensitive
Cefotaxime	Sensitive
Ceftazidime	Sensitive
Ceftriaxone	Sensitive
Ciprofloxacin	Sensitive
Gentamicin	Sensitive
Imipenem	Sensitive
Levofloxacin	Sensitive
Meropenem	Sensitive

Further history regarding the patient's diarrheal illness revealed that he had consumed a variety of seafood, including raw oysters, at a local restaurant. Subsequently, he developed diarrhea two days later. Due to his profuse diarrhea, the patient experienced fecal matter running down his legs, which included his exposed wound. This is likely the cause of his wound infection. With antibiotic therapy, the patient clinically improved and was discharged home to complete a 14-day course of doxycycline. The public health department was notified of this case. Throughout his hospitalization, he was observed to have pancytopenia. The patient mentioned that he had been experiencing pancytopenia for approximately one year. He underwent a bone marrow biopsy. The results showed a mildly hypercellular marrow with mild dyspoietic features. However, these findings are nonspecific, and the differential diagnosis remains broad, including conditions like iron deficiency anemia, acute infection, immune suppression, etc. Due to the splenomegaly detected on a CT scan of his abdomen and pelvis, along with the presence of pancytopenia, there was concern that an underlying bone marrow disorder might be responsible for his symptoms. As a result, the patient will be scheduled for outpatient follow-up with a local specialist for further workup.

## Discussion

Infection with *V. fluvialis *is often a self-limited case of gastroenteritis but can be complicated by bacteremia, wound infections, and cases of cerebritis, peritonitis, and otitis externa have also been reported. There have been very few case reports of bacteremia caused by *V. fluvialis*, with the first one reported in 2006 by Lai et al. [3]. In these case reports, all patients were immunocompromised. Due to the limited number of *V. fluvialis *bacteremia case reports and the absence of established guidelines, there is no specific duration set for monitoring cultures. However, considering our patient's blood cultures yielded *V. fluvialis* on the 13th day, we recommend monitoring the cultures for at least two weeks.

Huang and Hsu described a case of hemorrhagic cellulitis and cerebritis. The patient was immunocompromised due to excessive alcohol intake and was initially treated with oxacillin and gentamicin. The patient did not improve with this regimen and subsequently was transitioned to ceftazidime and oxytetracycline, with blood cultures ultimately showing sensitivity to ceftazidime. This case suggested prompt debridement of the hemorrhagic skin lesions, which was in contrast to this case where the patient improved with antibiotic therapy alone [4]. In our case, we believe the secondary infection of the patient's wounds with *V. fluvialis* prompted the development of hemorrhagic bullae.

Ratnaraja et al. reported a case where a patient had two episodes of *V. fluvialis* associated with peritoneal dialysis. The first episode was empirically treated with ceftriaxone, and susceptibilities ultimately showed it was “susceptible to ciprofloxacin, ceftriaxone, and imipenem but resistant to amoxicillin, amoxicillin-clavulanic acid, and gentamicin.” The second episode was initially treated with intraperitoneal gentamicin and transitioned to ciprofloxacin for 21 days. It was thought the patient developed these cases due to contaminated seafood [5].

Rodriguez et al reported a case of *V. fluvialis *otitis externa in an AIDS patient with a CD4 count of 123 cells/mm³ after swimming in a pool of seawater. The susceptibilities showed *V. fluvialis* was sensitive to tetracycline, ampicillin, chloramphenicol, trimethoprim-sulfamethoxazole, nalidixic acid, ciprofloxacin, streptomycin, erythromycin, gentamicin, cefuroxime, and polymyxin B. The patient was treated with a 10-day course of tetracycline [6].

The similarity in these cases of rare, unexpected extraintestinal manifestations of *V. fluvialis *is the presence of an immunocompromised state. Although there was no established diagnosis in this patient that would deem him immunocompromised, due to his history of pancytopenia and splenomegaly, we suspected he did have an underlying condition, such as myelofibrosis, that led him to be susceptible to systemic infection by *V. fluvialis*.

Necrotizing fasciitis was an additional concern in this patient, which, fortunately, did not develop. In a retrospective study of 70 patients with *V. fluvialis* necrotizing fasciitis, it was suggested that severe hypoalbuminemia, severe thrombocytopenia, and an increase in banded forms of leukocytes were potential signs of necrotizing fasciitis. The researchers proposed that these indicators may be more useful than the laboratory risk indicator for necrotizing fasciitis (LRINEC) scoring system in identifying cases of *V. fluvialis* necrotizing fasciitis [7].

Although there is no established guideline-directed therapy for *V.* *fluvialis*, cases of extraintestinal infections were often treated with third-generation cephalosporins, doxycycline, amoxicillin-clavulanate, fluoroquinolones, and tetracycline [1,6]. In this case, the bacterium was sensitive to all antibiotics tested, which included cephalosporins, ciprofloxacin, meropenem, imipenem, levofloxacin, and aztreonam.

## Conclusions

*V. fluvialis* is of growing concern in public health, and although the typical presentation is gastroenteritis, there are much more sinister manifestations that can develop, especially in individuals with compromised immune systems. A thorough dermatological examination is necessary, given the reported skin manifestations, such as hemorrhagic cellulitis, and, in this case, hemorrhagic bullae. There are currently no established guidelines for antibiotic therapy. We suggest monitoring the blood cultures for at least two weeks and tailoring antibiotics according to the susceptibility report. The patient in our case was successfully treated with doxycycline.
